# Melanoma and CLL co-occurrence and survival: role of KC history

**DOI:** 10.1186/s12885-023-11573-z

**Published:** 2023-11-09

**Authors:** Yayi Zhao, Rossybelle P. Amorrortu, Sandra C. Stewart, Kavita M. Ghia, Vonetta L. Williams, Vernon K. Sondak, Kenneth Y. Tsai, Javier Pinilla-Ibarz, Julio C. Chavez, Dana E. Rollison

**Affiliations:** 1 Department of Cancer Epidemiology, Moffitt Cancer Center, Tampa, FL USA; 2 Department of Cancer Registry, Moffitt Cancer Center, Tampa, FL USA; 3 Collaborative Data Services Core, Moffitt Cancer Center, Tampa, FL USA; 4 Department of Cutaneous Oncology, Moffitt Cancer Center, Tampa, FL USA; 5 Department of Anatomic Pathology, Moffitt Cancer Center, Tampa, FL USA; 6 Department of Malignant Hematology, Moffitt Cancer Center, Tampa, FL USA

**Keywords:** Keratinocyte carcinoma, Melanoma, Chronic lymphocytic leukemia, Survival, Cancer diagnosis

## Abstract

**Background:**

Survival following melanoma and chronic lymphocytic leukemia (CLL) have both been individually associated with previous history of non-melanoma skin cancers (specifically keratinocyte carcinomas [KC]). Furthermore, melanoma and CLL have been reported to occur within the same patients. The survival experience of patients with both cancers is understudied, and the role of history of KC is unknown. Additional research is needed to tease apart the independent associations between KC and CLL survival, KC and melanoma survival, and the co-occurrence of all three cancers.

**Methods:**

A retrospective cohort study was conducted among patients who were diagnosed with melanoma and/or CLL at a comprehensive cancer center between 2008 and 2020. Multivariable Cox regression models were used to examine the association between history of KC and survival following melanoma and/or CLL with careful consideration of calendar year of diagnosis, treatment regimens and other risk factors. A nested case–control study comparing patients with both CLL and melanoma to those with only CLL or only melanoma was conducted to compare blood parameters across the three groups.

**Results:**

A time-dependent association was observed between history of KC and favorable melanoma survival within 4 years following diagnosis and poorer survival post 7 years after melanoma diagnosis. History of KC was not significantly associated with survival following the diagnosis of CLL, after adjustment for clinical factors including historical/concurrent melanoma. Patients with co-occurring melanoma and CLL tended to be diagnosed with melanoma first and had elevated blood parameters including white blood cell and lymphocyte counts as compared with patients who were diagnosed with only melanoma.

**Conclusions:**

History of KC was an independent predictor of survival following melanoma but not of CLL. Additional studies are needed to determine if blood parameters obtained at the time of melanoma diagnosis could be used as a cost-effective way to identify those at high risk of asymptomatic CLL for the promotion of earlier CLL diagnosis.

**Supplementary Information:**

The online version contains supplementary material available at 10.1186/s12885-023-11573-z.

## Background

Previous studies have reported the co-occurrence of melanoma and chronic lymphocytic leukemia (CLL) within the same individuals [1–6] possibly due to shared risk factors. For example, a history of keratinocyte carcinomas (KC), which includes cutaneous squamous cell carcinoma (cuSCC) and basal cell carcinoma (BCC), has been hypothesized as a shared risk factor [7–12]. BCC and cuSCC are both associated with ultraviolet radiation (UVR) [13, 14] with BCC linked to intermittent and childhood UVR exposure and cuSCC linked to chronic sun exposure and immunosuppression [15, 16]. In our previous work, we observed that a history of KC occurred more often among patients with melanoma or CLL compared to patients with other malignancies including cancers of the breast, prostate, lung, colon and non-Hodgkin lymphoma [17]. Furthermore, a history of KC was associated with reduced survival following a melanoma or CLL diagnosis in this same study population [17]. Elucidation of prognostic markers can be useful for risk stratification and surveillance of subsequent melanoma and CLL. However, no previous studies have been able to tease apart independent effects of KC on melanoma and CLL survival due to lack of detailed information on cancer treatments and other clinical parameters.

We conducted a retrospective cohort study of patients treated for melanoma and CLL at Moffitt Cancer Center (MCC) in 2008–2020, to more fully characterize the associations between KC history, melanoma, and CLL. Factors including calendar year of diagnosis, tumor histology, specific treatment regimens, and other cancer risk factors were explored as both potential confounders and effect modifiers of the association between history of KC and survival following melanoma/CLL. In addition, a nested case–control analysis was conducted to compare blood parameters between patients with both melanoma and CLL to those with only one of these cancers.

## Methods

### Study design and population

Methods of this retrospective cohort study have been previously reported in detail [17]. Briefly, data from MCC patients who were diagnosed with melanoma or CLL at age 18 or older were identified to examine the association between KC history and the survival following either cancer type. The melanoma and CLL cohorts were defined using site codes from International Classification of Diseases for Oncology, 3^rd^ edition (ICD-O3) [18]. Patients were considered eligible for the study if they 1) were diagnosed and/or treated at MCC for melanoma or CLL between December 2008-April 2020, 2) completed the Electronic Patient Questionnaire (EPQ), a questionnaire administered to all patients new to MCC, and 3) had a non-missing response to the personal history of cancer question in the EPQ. In total 5,511 and 571 patients were included in the melanoma and CLL cohorts, respectively.

An exploratory case–control analysis nested within the larger cohort was conducted to further investigate the co-occurrence of melanoma and CLL. The subgroup of patients diagnosed with both cancers who had available blood parameters (*n* = 17) were identified and compared with two matched control groups: a) patients who were diagnosed with only melanoma (melanoma controls), and b) patients who were diagnosed with only CLL (CLL controls). The comparisons of blood parameters were limited to a subset of 13 cases with blood parameters available within ± 60 days of their initial cancer diagnosis date, and the matched controls including: 26 melanoma controls (1:2 ratio) and 13 CLL controls (1:1 ratio, as it was not possible to identify more than 1 matched control for each case).

### Data collection

Patient characteristics were extracted from the MCC Cancer Registry and the electronic health record (EHR) including a) clinical factors: date of diagnosis, tumor histology, tumor stage at diagnosis, first course treatments including chemotherapy (both cytotoxic chemotherapy and molecularly targeted agents), immunotherapy and radiation, therapeutic drugs used as first course treatment, Breslow thickness (melanoma cohort only), historical and concurrent melanoma (identified using ICD diagnosis codes), and b) sociodemographic and other factors: age, sex, race, ethnicity, payment methods, date of last contact, and associated vital status. Self-reported data on smoking, alcohol use, history of autoimmune disease, history of KC, family history of melanoma, and aspirin use in the last year were obtained from the EPQ. Among the sub-cohort of patients who were included in the nested case–control analysis, blood parameters closest to the corresponding cancer diagnosis were extracted from EHR. The following blood parameters were included in the analysis based on their diagnostic potential for CLL and role as markers of systemic inflammation (white blood cell [WBC] count, platelet count, hematocrit, hemoglobin, lymphocyte count, monocyte count, neutrophil count, neutrophil band, and polyploid neutrophil) [19].

### Statistical analysis

Patient demographic and clinical characteristics were presented separately for the melanoma and CLL cohorts. Venn diagrams were used to describe the combinations of first course treatment and 5-year survival rates for melanoma including radiation, chemotherapy, and immunotherapy with respect to stage at diagnosis and calendar year of diagnosis. Similar methods were used to visualize the combination of treatments received by the CLL cohort, except for radiation therapy due to its infrequent use.

Based on the previously observed time-varying association between history of KC and survival following melanoma [17], a time-dependent Cox regression model with a backward elimination process was used to derive a final model. This modeling process started with a full list of covariates including age at diagnosis, sex, race, ethnicity, year of diagnosis, stage at diagnosis, smoking status, alcohol use, family history of melanoma, first course treatment of radiation, immunotherapy and chemotherapy, history of autoimmune disease, aspirin use, Breslow thickness, and insurance status. Then, the backward elimination procedure dropped the factor with the largest p-value at each run until all factors remaining in the model showed p-values smaller than 0.05. Subsequently, the final melanoma model was stratified by key factors that could potentially modify the association with KC history, including calendar year of diagnosis, tumor histology, stage at diagnosis, and first course treatment.

A similar modeling process was used to estimate the association between KC history and survival following a CLL diagnosis without a time-varying association. The following variables were included in the CLL multivariate model: age at diagnosis, sex, smoking status, historical and concurrent melanoma, first course treatment of radiation, immunotherapy and chemotherapy, insurance status, and self-reported history of melanoma. The analysis was stratified by calendar year of diagnosis and first course treatment.

To better understand whether specific medications affect the association between history of KC and survival following melanoma, the most frequently used chemotherapy and immunotherapy medications were analyzed. Unadjusted Cox regression models were used to estimate the association within each subgroup of patients who received each specific type of medication given the small sample size. A similar approach was used to evaluate potential effect modification by chemotherapy and immunotherapy medications among CLL patients.

In the nested case–control analysis, matched random sampling was performed to identify two groups of controls with respect to age at diagnosis in decades, sex, calendar year at diagnosis, smoking status, and Breslow thickness and stage at diagnosis (for melanoma only). Patient demographic and clinical characteristics were reported for the case group and the two matched control groups. The blood parameters were compared between the case group and the matched control groups using paired Wilcoxon sign-rank test with adjustment for multiple comparison using the false discovery rate method. Subsequently, a sensitivity analysis was conducted to exclude 3 cases (and their corresponding matched controls) whose CLL was diagnosed prior to their melanoma diagnosis.

## Results

Among patients with melanoma, the mean age at diagnosis was 62 years, 60% of the patients were male, 99% were White race, and 98% were non-Hispanic. CLL patients had a mean age of 63 years, 63% were male, 93% were White race, and 95% were non-Hispanic. Prevalence of KC history was 29% among melanoma patients and 20% among CLL patients. Further, history of KC was most prevalent among those who were diagnosed with melanoma between 2012–2020 as compared to those who were diagnosed with melanoma prior to 2012, with a similar pattern observed among CLL patients. Approximately 40% of the patients diagnosed with desmoplastic or lentigo melanoma reported a history of KC, while approximately 25% of melanoma patients diagnosed with other histologic types reported a history of KC (Table 1).

**Table 1:** Baseline demographic and clinical characteristics among patients diagnosed with melanoma or chronic lymphocytic leukemia (CLL)

Characteristics	Melanoman (%)		CLLn (%)	
	**All patients**	**Patients with KC history**	**All patients**	**Patients with KC history**
**Total sample size**	5511	1579 (28.7)	571	113 (19.8)
**Age at diagnosis**				
mean (SD)	62.0 (15.1)	68.4 (11.6)	63.3 (11.2)	69.6 (9.0)
**Age at diagnosis (categorized)**				
18–29	168 (3.0)	6 (0.4)	2 (0.4)	0 (0)
30–39	363 (6.6)	23 (1.5)	15 (2.6)	0 (0)
40–49	578 (10.5)	70 (4.4)	41 (7.2)	2 (1.8)
50–59	1015 (18.4)	228 (14.4)	149 (26.1)	15 (13.3)
60–69	1481 (26.9)	467 (29.6)	177 (31.0)	31 (27.4)
70–79	1298 (23.6)	527 (33.4)	154 (27.0)	53 (46.9)
80 and above	608 (11.0)	258 (16.3)	33 (5.8)	12 (10.6)
**Calendar year at diagnosis**^**a**^				
2009–2011/2009–2012	792 (14.4)	149 (9.4)	129 (22.6)	12 (10.6)
2012–2015/2013–2015	2184 (39.6)	654 (41.4)	144 (25.2)	33 (29.2)
2016–2020	2535 (46.0)	776 (49.1)	298 (52.2)	68 (60.2)
**Sex**				
Female	2187 (39.7)	529 (33.5)	209 (36.6)	33 (29.2)
Male	3324 (60.3)	1050 (66.5)	362 (63.4)	80 (70.8)
**Race**				
American Indian	3 (0.1)	1 (0.1)	0 (0)	0 (0)
Asian	8 (0.1)	1 (0.1)	5 (0.9)	0 (0)
Black	26 (0.5)	1 (0.1)	21 (3.7)	0 (0)
White	5451 (98.9)	1571 (99.5)	532 (93.2)	113 (100.0)
Multiple races	9 (0.2)	3 (0.2)	5 (0.9)	0 (0)
Other	8 (0.1)	0 (0)	6 (1.1)	0 (0)
Unknown	6 (0.1)	2 (0.1)	2 (0.4)	0 (0)
**Ethnicity**				
Non-Spanish or Hispanic origin	5402 (98.0)	1565 (99.1)	540 (94.6)	111 (98.2)
Spanish or Hispanic origin	105 (1.9)	14 (0.9)	31 (5.4)	2 (1.8)
Unknown	4 (0.1)	0 (0)		
**Histology**^**b**^				
Desmoplastic Melanoma	312 (5.7)	130 (8.2)		
Lentigo Melanoma	341 (6.2)	139 (8.8)		
Melanoma Nodular	794 (14.4)	186 (11.8)		
Superficial Spreading Melanoma	2479 (45.0)	705 (44.6)		
Other	1585 (28.8)	419 (26.5)		
**SEER stage at presentation**^**c**^				
In situ	3 (0.1)	0 (0)		
Localized	3911 (71.0)	1201 (76.1)		
Regional	1131 (20.5)	286 (18.1)		
Distant metastasis	408 (7.4)	83 (5.3)		
Unknown/NA	58 (1.1)	9 (0.6)		
**AJCC stage at diagnosis**^**c**^				
0	15 (0.3)	7 (0.4)		
1	2954 (53.6)	900 (57.0)		
2	1149 (20.8)	362 (22.9)		
3	973 (17.7)	223 (14.1)		
4	333 (6.0)	65 (4.1)		
Unknown/NA	87 (1.6)	22 (1.4)		

^a^Cut-offs for calendar year at diagnosis were determined based on changes in treatment of melanoma and CLL

^b^Histology data are not presented for CLL as they were all mature non-Hodgkin lymphoma B-cell

^c^American Joint Committee on Cancer (AJCC) and Surveillance, Epidemiology and End Results (SEER) stage data are not presented for CLL as they do not follow the same staging systems as solid tumors

Changes in treatment were observed by stage and calendar year for patients diagnosed with melanoma. For example, among those diagnosed with stage 3 or 4 melanoma, the use of chemotherapy alone decreased from 22 to 8% while the use of only immunotherapy increased from 35 to 65% between 2009–2020 (Additional file 1). For patients diagnosed with CLL, a shift was observed from using both chemotherapy and immunotherapy to using only immunotherapy (Additional file 2).

The associations between history of KC and survival following melanoma/CLL were examined in the context of sociodemographic and clinical factors. KC history had a time-dependent effect on survival following a melanoma diagnosis, after controlling for age at diagnosis, sex, calendar year of diagnosis, stage at diagnosis, history of smoking, alcohol use, chemotherapy treatment, history of autoimmune disease, Breslow thickness, and insurance status (Table 2). Melanoma patients with a history of KC experienced favorable survival within 4 years of a melanoma diagnosis (hazard ratio [HR] [95% confidence interval (CI)] = 0.75 [0.62–0.91]), no differences in survival between 5–7 years following a melanoma diagnosis (HR [95%CI] = 1.04 [0.73–1.47]), and poorer survival following 7 or more years since the melanoma diagnosis (HR [95% CI] = 2.79 [1.44–5.41]) as compared to patients without a history of KC (Table 2). A similar time-dependent association between history of KC and survival following a melanoma diagnosis was consistently observed between subgroups of patients as defined by calendar year at diagnosis, tumor histology, and first course treatment (Additional file 3). No differences were observed in the association between history of KC and survival following the first 4 years of melanoma diagnosis by the type of chemotherapy (Additional file 4). However, some inconsistent patterns were found by specific types of immunotherapies (Additional file 4).

**Table 2:** Association between history of keratinocyte carcinoma and survival following diagnosis of melanoma

**Model factors**		
	**n (%)**	**Hazard ratio (95% confidence interval)**^**a**^
**Age at diagnosis** (mean [SD])	62.0 (15.1)	1.04 (1.03–1.05)
**Sex**		
Female	2187 (39.7)	1.0 (ref.)
Male	3324 (60.3)	1.42 (1.21–1.67)
**Ethnicity**		
Non-Spanish or Hispanic Origin	5402 (98.1)	1.0 (ref.)
Spanish or Hispanic Origin	105 (1.9)	1.88 (1.21–2.93)
**Year of diagnosis**		
2009–2011	792 (14.4)	1.0 (ref.)
2012–2015	2184 (39.6)	0.75 (0.63–0.89)
2016–2020	2535 (46.0)	0.62 (0.49–0.78)
**Stage at diagnosis**		
Stage 1	2954 (54.6)	1.0 (ref.)
Stage 2	1149 (21.2)	1.77 (1.38–2.28)
Stage 3	973 (18.0)	3.13 (2.49–3.94)
Stage 4	333 (6.2)	8.57 (6.35–11.56)
**Smoking status**		
Never	2686 (50.0)	1.0 (ref.)
Former	2181 (40.6)	1.22 (1.04–1.43)
Current	505 (9.4)	2.34 (1.85–2.97)
**Drank in the last year**		
No	1294 (23.7)	1.0 (ref.)
Yes	4174 (76.3)	0.71 (0.60–0.83)
**Chemotherapy as first course treatment**		
No	5365 (97.4)	1.0 (ref.)
Yes	146 (2.6)	2.93 (2.08–4.13)
**History of autoimmune disease**		
No	4768 (94.8)	1.0 (ref.)
Yes	259 (5.2)	1.42 (1.06–1.91)
**Breslow thickness**		
Less than 1 mm	2058 (41.7)	1.0 (ref.)
Between 1 and 2 mm	1315 (26.6)	1.38 (1.11–1.71)
Above 2 mm	1562 (31.7)	1.60 (1.27–2.02)
**Insurance status**		
Private/Manage	2364 (44.6)	1.0 (ref.)
Self Pay/Not Insured	219 (4.1)	1.06 (0.72–1.57)
Medicaid	82 (1.5)	1.45 (0.90–2.33)
Medicare	2478 (46.7)	1.41 (1.13–1.76)
Tricare/VA	160 (3.0)	1.72 (1.12–2.63)
**History of KC: time since melanoma diagnosis**^**b**^		
History of KC: within first 4 years	1579 (28.7)^2a^	0.75 (0.62–0.91)
History of KC: between 5–7 years	661 (26.7)^2b^	1.04 (0.73–1.47)
History of KC: more than 7 years	211 (23.6)^2c^	2.79 (1.44–5.41)

^a^The Cox regression model was derived using a backward elimination process on a full model including the following factors: **age at diagnosis**, **sex**, race, **ethnicity**, **year of diagnosis**, **stage at diagnosis**, **smoking status**, **drank in the last year**, family history of melanoma, first course treatment of radiation, immunotherapy and **chemotherapy**, **history of autoimmune disease**, use of aspirin in the last year, **Breslow thickness**, **insurance status**, and **history of keratinocyte carcinoma** (bolded variables were retained in the final model)

^b^The frequency and percentage of patients who self-reported history of keratinocyte carcinoma among the ^2a^full cohort, among ^2b^patients who had at least 4 years of follow-up, and among ^2c^patients who had at least 7 years of follow-up

The risk of death following CLL was elevated among patients who reported a history of KC (HR = 1.53, 95% CI = 0.95–2.45), after adjustment for age at diagnosis, smoking status, any melanoma diagnosis, and chemotherapy (Table 3). This pattern remained consistent across subgroups of patients who received immunotherapy or chemotherapy but showed directional difference when investigating specific medications/regimens (Additional file 5 and 6). The association between KC and survival following CLL was also inconsistent across subgroups of patients by calendar year of diagnosis (Additional file 5).

**Table 3:** Association between history of keratinocyte carcinoma and survival following diagnosis of chronic lymphocytic leukemia

**Model factors**		
	**n (%)**	**Hazard ratio (95% confidence interval)**^**a**^
**Age at diagnosis** (mean [SD])	63.3 (11.2)	1.07 (1.04–1.09)
**Smoking status**		
Never	286 (51.0)	1.0 (ref.)
Former	246 (43.9)	1.61 (1.03–2.50)
Current	29 (5.2)	3.2 (1.28–8.01)
**Historical or concurrent melanoma (based on ICD billing codes)**		
No	536 (93.9)	1.0 (ref.)
Yes	35 (6.1)	2.11 (1.13–3.95)
**Chemotherapy as first course treatment**		
No	480 (84.1)	1.0 (ref.)
Yes	91 (15.9)	2.96 (1.90–4.61)
**History of KC**		
No	458 (80.2)	1.0 (ref.)
Yes	113 (19.8)	1.53 (0.95–2.45)

^a^The Cox regression model was derived using a backward elimination process on a full model including the following factors: **age at diagnosis**, sex, **smoking status**, **historical or concurrent melanoma (based on ICD billing codes)**, first course treatment of radiation, immunotherapy and **chemotherapy**, insurance status, self-reported history of melanoma, and history of keratinocyte carcinoma (bolded variables were retained in the final model)

**Fig. 1:**
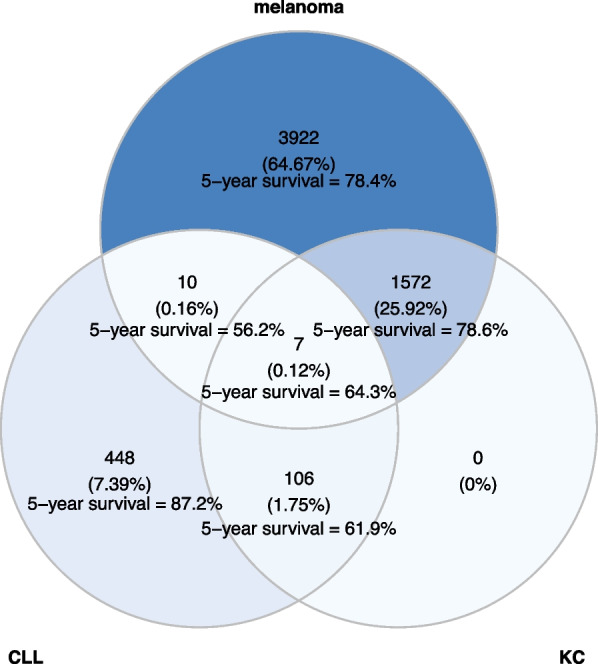
Diagnosis of melanoma and chronic lymphocytic leukemia (CLL), and history of keratinocyte carcinoma (KC). Venn diagram of groups of patients as defined by history of keratinocyte carcinoma (KC) and diagnosis of melanoma and/or chronic lymphocytic leukemia (CLL). Patients with both melanoma and CLL were more likely to have a history of KC (41%) compared to patients diagnosed with only CLL (19%) or patients diagnosed with only melanoma (29%)

Patients with both melanoma and CLL were more likely to have a history of KC (41%) compared to patients diagnosed with CLL who did not have melanoma (19%) or patients diagnosed with melanoma who did not have CLL (29%) (Fig. 1). In addition, patients diagnosed with CLL without skin cancer had a higher 5-year survival rate (87.2%) than CLL patients with skin cancer (~ 60%) regardless of whether the skin cancer was melanoma or KC. Given these results, we conducted an exploratory nested case–control study to investigate the co-occurrence of melanoma and CLL. Among the 52 patients included in the analysis, the mean age at the time of cancer diagnosis was 75, with males accounting for 92% of the sub-sample (Table 4). Ten of the 13 patients diagnosed with both cancers had a melanoma diagnosed prior to the CLL, among which 9 patients had CLL diagnosed within 60 days after the melanoma diagnosis. The WBC counts were significantly higher among cases (mean [SD] = 15.67 [12.33]) as compared with melanoma controls (mean [SD] = 9.57 [7.52]) (Table 4) with similar findings observed for lymphocyte counts (Δmean = 6.46) and percent lymphocytes (Δmean = 25.08). These comparisons remained significant in a sensitivity analysis where patients diagnosed with CLL prior to the melanoma were excluded. No other blood parameters differed between the cases and melanoma controls. Further, no significant differences were found when comparing the blood parameters between the cases and the CLL controls (Table 5).

**Table 4:** Baseline demographic and clinical characteristics among cases, melanoma controls, and chronic lymphocytic leukemia (CLL) controls

Demographical and clinical characteristics	Cases n (%)	CLL controls n (%)	Melanoma controls n (%)
**Total sample size**	13	13	26
**Age at melanoma diagnosis**			
mean (SD)	75.0 (5.8)		73.5 (7.7)
60–69	4 (30.8)		8 (30.8)
70–79	5 (38.5)		10 (38.5)
80 and above	4 (30.8)		8 (30.8)
**Age at CLL diagnosis**			
mean (SD)	75.2 (5.5)	74.5 (7.0)	
60–69	2 (15.4)	4 (30.8)	
70–79	7 (53.8)	5 (38.5)	
80 and above	4 (30.8)	4 (30.8)	
**Sex**			
Female	1 (7.7)	1 (7.7)	2 (7.7)
Male	12 (92.3)	12 (92.3)	24 (92.3)
**Race**			
White	13 (100.0)	12 (92.3)	26 (100.0)
Other	0 (0.0)	1 (7.7)	0 (0.0)
**Ethnicity**			
Non-Spanish or Hispanic origin	12 (92.3)	11 (84.6)	26 (100.0)
Spanish or Hispanic origin	1 (7.7)	2 (15.4)	0 (0.0)
**AJCC stage at diagnosis**^**a**^			
1	2 (15.4)		4 (15.4)
2	6 (46.2)		12 (46.2)
3	4 (30.8)		8 (30.8)
4	1 (7.7)		2 (7.7)

^a^American Joint Committee on Cancer (AJCC) stage data are not presented for CLL as they do not follow the same staging systems as solid tumors

**Table 5:** Blood parameters among groups of patients defined by diagnoses of melanoma and/or chronic lymphocytic leukemia

Blood parameter	Comparison between					
	Cases	melanoma controls	*p*-value^1^	Cases	CLL controls	*p*-value^1^
**White blood cell (k/uL)**						
n	13	26		13	13	
mean (SD)	15.67 (12.33)	9.57 (7.52)	0.010	15.67 (12.33)	16.99 (11.45)	0.888
**Platelet count (k/uL)**						
n	13	26		13	13	
mean (SD)	199.23 (36.54)	218.27 (110.20)	0.839	199.23 (36.54)	204.31 (84.09)	0. 972
**Hematocrit (%)**						
n	13	26		13	13	
mean (SD)	42.52 (3.61)	41.01 (5.60)	0.538	42.52 (3.61)	42.06 (3.59)	0.972
**Hemoglobin (g/dL)**						
n	13	26		13	13	
mean (SD)	14.36 (1.24)	13.72 (1.96)	0.343	14.36 (1.24)	13.88 (1.30)	0. 888
**Lymphocyte count (k/uL)**						
n	9	16		9	9	
mean (SD)	8.26 (12.63)	1.80 (1.03)	< 0.001	8.26 (12.63)	9.21 (7.08)	0. 888
Percent lymph—mean (SD)	47.99 (21.71)	22.91 (9.15)	< 0.001	47.99 (21.71)	53.83 (26.91)	0. 888
**Monocyte count (k/uL)**						
n	9	16		9	9	
mean (SD)	0.60 (0.26)	0.65 (0.24)	0.488	0.60 (0.26)	0.69 (0.37)	0. 888
**Neutrophil count (k/uL)**						
n	5	8		4	4	
mean (SD)	3.83 (1.00)	4.69 (2.14)		3.61 (1.01)	6.12 (3.72)	
Percent Neutrophil—mean (SD)	58.46 (8.21)	67.19 (8.86)		58.25 (9.46)	61.83 (13.01)	
**Neutrophil band (k/uL)**						
n	1	1		3	3	
mean (SD)	0.00 (NA)	0.07 (NA)		0.00 (0.00)	0.00 (0.00)	
Percent neutrophil band—mean (SD)	0.00 (NA)	1.00 (NA)		0.00 (0.00)	0.00 (0.00)	
**Polyploid neutrophil (k/uL)**						
n	2	3		4	4	
mean (SD)	3.84 (0.59)	9.37 (7.06)		3.78 (0.94)	4.94 (3.43)	
Percent polyploid neutrophil—mean (SD)	29.50 (6.36)	66.67 (22.85)		23.50 (9.95)	23.75 (13.07)	

^1^*P*-values were calculated using paired Wilcoxon sign-rank test for parameters that were available among at least 5 cases and were adjusted for multiple comparison using false discovery rate method.

## Discussion

After adjustment for multiple patient factors, we observed a significant inverse association between history of KC and melanoma survival within the first 4 years since diagnosis. However, patients with a history of KC were found to have worse overall survival post 7 years of a melanoma diagnosis. This time-dependent association is consistent with our previous observations in an age- and stage-adjusted model, [17] suggesting that the association between history of KC and melanoma survival is independent of clinical factors including melanoma stage, Breslow thickness, history of autoimmune disease, and first course treatment. In addition, the current analysis found no difference in the association between history of KC and melanoma when stratifying by types of first course treatment. There is no clear biological explanation for the observed time-dependent associations, and if they are similarly observed in other study populations, additional research into underlying mechanism is warranted.

A previous study reported an association between history of KC and worse survival following CLL diagnosis [8]. However, a history of KC was not significantly associated with survival following CLL in this analysis after adjustment for demographic and clinical factors. Additionally, the corresponding hazard ratio in the current study (HR = 1.53) was lower than the age- and sex-adjusted model (HR = 1.73) reported in our previous analysis [17] but larger than a previous study that examined CLL survival (HR = 1.29) [8] and two studies that examined non-Hodgkin lymphoma/CLL survival (HRs = 1.33/1.32) [20, 21]. The loss of statistical significance in our current analysis could be due to limited sample size or the adjustment for additional factors (e.g., historical/concurrent melanoma), which potentially serve as confounders in the association between history of KC and CLL survival. Since KC and melanoma share common risk factors including age, Fitzpatrick skin phototypes 1–3 (e.g., light skin/hair) [10], exposure to ultraviolet radiation, and immunosuppression [11, 12], it is likely that adjustment for melanoma reduced the effect estimates for the association between KC history and CLL survival. In fact, in a sensitivity analysis, a history of KC was significantly associated with CLL survival when the melanoma covariable was removed from the original multivariable model (HR = 1.70, 95% CI = 1.08–2.69). Together, these results suggest that the association between KC history and survival following CLL was confounded by melanoma at a magnitude of 11.1%. Collectively, KC and melanoma may predict worse survival following CLL through a shared biologic pathway such as immune dysregulation and/or DNA repair [22–25].

Patients diagnosed with both melanoma and CLL had a higher prevalence of KC history as compared to patients diagnosed with only melanoma or CLL. CLL is known to increase the susceptibility and incidence of cutaneous malignancies including KC and melanoma [2, 3, 26–30]. However, in our study, most patients diagnosed with both cancers were diagnosed with melanoma prior to CLL. Although the biological mechanism is not fully understood, the severe T-cells immunosuppression induced by the CLL cells maybe one of the key aspects to this increase in incidence [29–31]. Such immunosuppression occurs even in the earliest phases of the disease, before the CLL is clinically identified – which frequently occurs during the evaluation and treatment for melanoma [1, 19]. Within the nested case–control analysis, we found that WBC and lymphocyte counts were elevated among patients diagnosed with both cancers (cases) as compared to their melanoma controls. These findings suggest that CLL was likely an incidental finding during the workup of melanoma. The elevated blood parameters thus pointed to undiagnosed CLL which is consistent with previous studies [1, 19]. Furthermore, when comparing the cases with CLL controls, no differences in the blood parameters were found, suggesting that the cases were not biologically distinct from the controls who were diagnosed with CLL alone.

Our study has several strengths worth noting. This study utilized patient data synthesized across multiple sources including EPQ, Cancer Registry, and the EHR to describe treatment, survival, demographic, and clinical factors for patients diagnosed with melanoma or CLL and to estimate the association between KC history and survival following these cancers. In addition, our study was the first to explore and confirm that the time-dependent effect of KC history on melanoma survival was consistent across calendar year of diagnosis, tumor histology, and first course treatment. Specifically, our results confirmed that a significant protective association exists between history of KC and survival within 4 years of melanoma diagnosis which is important as survival within a shorter time-period following diagnosis is more likely to approximate cancer-specific survival. Incorporating data for both cancers also allowed us to identify a unique sub-population that showed a higher prevalence of KC history. Finally, findings from the nested case–control analysis point to the importance of blood workup among melanoma patients, as specific blood parameters such as WBC and lymphocyte counts could serve as indicators of asymptomatic CLL, potentially leading to earlier diagnosis and risk stratification that may improve overall outcomes [32].

The study is not without limitations. First, as an observational study, our analysis was unable to directly establish causality [33, 34] and did not investigate biological mechanism for the observed associations. Due to the incomplete data on cause of death, we were unable to assess cancer-specific survival to examine the long-term effect from KC. Further, given that KC is not a reportable cancer to cancer registries, we were unable to ascertain KC occurring after the diagnosis of the CLL/melanoma and could not investigate the use of newly diagnosed KC as a potential method of risk stratification, although previous studies have found that patients with CLL have a up to a fivefold skin cancer risk following diagnosis of CLL [35]. The exploratory case–control analysis was limited by sample size and the availability of data on the blood parameters. However, sensitivity analyses indicated there were no differences in demographic or cancer characteristics between the patients with and without blood parameters, minimizing the possibility of selection bias for the case–control analyses. Future studies should seek to replicate these findings in larger study populations, possible through multi-center collaborations, given the rarity of melanoma and CLL co-occurrence [36]. Such future studies should consider investigating additional parameters such as lymphocyte subsets (CD4 T-cells and B-cells) and immunoglobulin G levels, which are more specific to immunosuppression for the purpose of identifying asymptomatic CLL among melanoma patients [31, 37].

## Conclusions

In summary, after adjustment for demographic and relevant clinical factors, a significant time-dependent association was found between history of KC and survival following diagnosis of melanoma. The overall survival following a CLL diagnosis was poorer among those with a history of KC, though not statistically significant. The nested case–control findings highlight the importance of blood workup among melanoma patients as specific blood parameters such as WBC and lymphocyte counts may signal asymptomatic CLL among melanoma patients to promote earlier diagnosis of CLL. Although an earlier diagnosis of CLL is not associated with better CLL-specific outcomes and, in most cases, won’t require any therapeutic intervention at diagnosis [36, 38], it may be helpful to counsel patients regarding the increased risk of subsequent malignancies and infections due to cancer-related immunosuppression. Future studies are needed to investigate whether our findings reflect a true impact of immunosuppression from CLL, or if they are driven by the increased presence of neoantigens from UVR exposure.

### Supplementary Information

**Additional file 1. **Radiation, chemotherapy, and immunotherapy as first course treatments received by melanoma patients, stratified by stage and calendar year at diagnosis. This file describes the combinations of treatment modalities received by melanoma patients who were diagnosed with earlier stage (stage 1 or 2) or later stage (stages 3 or 4) melanoma in 2009-2011, in 2012-2015, or in 2016-2020. 

**Additional file 2. **Chemotherapy and immunotherapy as first course treatments received by chronic lymphocytic leukemia (CLL) patients, stratified by calendar year at diagnosis. This file describes the combinations of treatment modalities received by patients diagnosed with chronic lymphocytic leukemia in 2009-2012, in 2013-2015, or in 2016-2020.

**Additional file 3. **Association between history of keratinocyte carcinoma (KC) and survival following diagnosis of melanoma, stratified by patient characteristics.This figure depicts the association between history of KC and survival following melanoma among different patient groups as defined by calendar year of diagnosis, melanoma histology, stage of melanoma, and types of first course treatment.

**Additional file 4. **Association between history of keratinocyte carcinoma (KC) and survival following melanoma diagnosis among patients receiving specific chemotherapy/immunotherapy medications. This figure depicts the association between history of KC and survival following melanoma among groups of patients as defined by the type of chemotherapy or the type of immunotherapy used as a part of first course treatment. 

**Additional file 5. **Association between history of keratinocyte carcinoma (KC) and survival following diagnosis of chronic lymphocytic leukemia (CLL), stratified by patient characteristics. This figure depicts the association between history of KC and survival following chronic lymphocytic leukemia among groups of patients as defined by calendar year of diagnosis and type of first course treatment.

**Additional file 6. **Association between history of keratinocyte carcinoma (KC) and survival following chronic lymphocytic leukemia (CLL) diagnosis among patients receiving specific chemotherapy/immunotherapy medications. This figure depicts the association between history of KC and survival following chronic lymphocytic leukemia among groups of patients as defined by the type of chemotherapy or the type of immunotherapy used as a part of first course treatment.

## Data Availability

The datasets presented in this article are not readily available in order to protect participant confidentiality and privacy. Requests to access the datasets should be directed to the corresponding author.

## Abbreviations

AJCC: American Joint Committee on Cancer
BCC: Basal cell carcinoma
CI: Confidence interval
CLL: Chronic lymphocytic leukemia
cuSCC: Cutaneous squamous cell carcinoma
EHR: Electronic Health Record
EPQ: Electronic Patient Questionnaire
HR: Hazard ratio
ICD-O3: International Classification of Diseases for Oncology, 3rd edition
KC: Keratinocyte carcinoma
MCC: Moffitt Cancer Center
NHL: Non-Hodgkin lymphoma
SD: Standard deviation
SEER: Surveillance, Epidemiology and End Results
UVR: Ultraviolet radiation
WBC: White blood cell
