# Supplementary material for: Melanoma and CLL co-occurrence and survival: role of KC history
Source: BMC Cancer. 2023 Nov 9;23:1084. doi: 10.1186/s12885-023-11573-z (PMC10636833; doi:10.1186/s12885-023-11573-z)
Supplement: Supplementary file 1 — Additional file 1. Radiation, chemotherapy, and immunotherapy as first course treatments received by melanoma patients, stratified by stage and calendar year at diagnosis. This file describes the combinations of treatment modalities received by melanoma patients who were diagnosed with earlier stage (stage 1 or 2) or later stage (stages 3 or 4) melanoma in 2009-2011, in 2012-2015, or in 2016-2020. [file 12885_2023_11573_MOESM1_ESM.docx]

**Additional file 1. Radiation, chemotherapy, and immunotherapy as first course treatments received by melanoma patients, stratified by stage and calendar year at diagnosis.**

| Treatment modality combinations | Diagnosed in 2009-2011 | |  | Diagnosed in 2012-2015 | |  | Diagnosed in 2016-2020 | |
| --- | --- | --- | --- | --- | --- | --- | --- | --- |
|  | patients n (%) | 5-year survival (%) |  | patients n (%) | 5-year survival (%) |  | patients n (%) | 5-year survival (%) |
| *Patients diagnosed with stage 1 or 2 melanoma* | |  |  |  |  |  |  |  |
| radiation only | 16 (76.2) | 56.2 |  | 46 (83.6) | 84.5 |  | 67 (69.1) | 71.3 |
| chemotherapy only | 0 (0) |  |  | 2 (3.6) | 100.0 |  | 3 (3.1) | 66.7 |
| immunotherapy only | 5 (23.8) | 80.0 |  | 6 (10.9) | 83.3 |  | 24 (24.7) | 90.4 |
| radiation and chemotherapy only | 0 (0) |  |  | 0 (0) |  |  | 0 (0) |  |
| radiation and immunotherapy only | 0 (0) |  |  | 1 (1.8) | 100.0 |  | 3 (3.1) | 100.0 |
| chemotherapy and immunotherapy only | 0 (0) |  |  | 0 (0) |  |  | 0 (0) |  |
| all three treatment modalities | 0 (0) |  |  | 0 (0) |  |  | 0 (0) |  |
|  |  |  |  |  |  |  |  |  |
| *Patients diagnosed with stage 3 or 4 melanoma* | |  |  |  |  |  |  |  |
| radiation only | 31 (29.8) | 35.5 |  | 62 (28.1) | 59.4 |  | 19 (5.7) | 44.3 |
| chemotherapy only | 23 (22.1) | 13.0 |  | 26 (11.8) | 23.1 |  | 25 (7.5) | 70.2 |
| immunotherapy only | 36 (34.6) | 58.3 |  | 90 (40.7) | 66.7 |  | 217 (64.8) | 77.1 |
| radiation and chemotherapy only | 5 (4.8) | 20.0 |  | 9 (4.1) | 33.3 |  | 9 (2.7) | 29.6 |
| radiation and immunotherapy only | 3 (2.9) | 66.7 |  | 17 (7.7) | 64.7 |  | 51 (15.2) | 48.5 |
| chemotherapy and immunotherapy only | 3 (2.9) | 0.0 |  | 15 (6.8) | 26.7 |  | 9 (2.7) | 40.6 |
| all three treatment modalities | 3 (2.9) | 33.3 |  | 2 (0.9) | 0.0 |  | 5 (1.5) | 0.0 |
