# Supplementary material for: Melanoma and CLL co-occurrence and survival: role of KC history
Source: BMC Cancer. 2023 Nov 9;23:1084. doi: 10.1186/s12885-023-11573-z (PMC10636833; doi:10.1186/s12885-023-11573-z)
Supplement: Supplementary file 2 — Additional file 2. Chemotherapy and immunotherapy as first course treatments received by chronic lymphocytic leukemia (CLL) patients, stratified by calendar year at diagnosis. This file describes the combinations of treatment modalities received by patients diagnosed with chronic lymphocytic leukemia in 2009-2012, in 2013-2015, or in 2016-2020. [file 12885_2023_11573_MOESM2_ESM.docx]

**Additional file 2. Chemotherapy and immunotherapy as first course treatments received by chronic lymphocytic leukemia (CLL) patients, stratified by calendar year at diagnosis.**

| Treatment modality combinations | Diagnosed in 2009-2012 | |  | Diagnosed in 2013-2015 | |  | Diagnosed in 2016-2020 | |
| --- | --- | --- | --- | --- | --- | --- | --- | --- |
|  | patients n (%) | 5-year survival (%) |  | patients n (%) | 5-year survival (%) |  | patients n (%) | 5-year survival (%) |
| chemotherapy only | 2 (6.1) | 100.0 |  | 6 (25.0) | 33.3 |  | 24 (50.0) | 65.5 |
| immunotherapy only | 3 (9.1) | 66.7 |  | 5 (20.8) | 80.0 |  | 6 (12.5) | 100.0 |
| both chemotherapy and immunotherapy | 28 (84.9) | 67.9 |  | 13 (54.2) | 49.2 |  | 18 (37.5) | 63.0 |
