# Supplementary material for: Melanoma and CLL co-occurrence and survival: role of KC history
Source: BMC Cancer. 2023 Nov 9;23:1084. doi: 10.1186/s12885-023-11573-z (PMC10636833; doi:10.1186/s12885-023-11573-z)

**Additional file 3. Association between history of keratinocyte carcinoma (KC) and survival following diagnosis of melanoma, stratified by patient characteristics.**


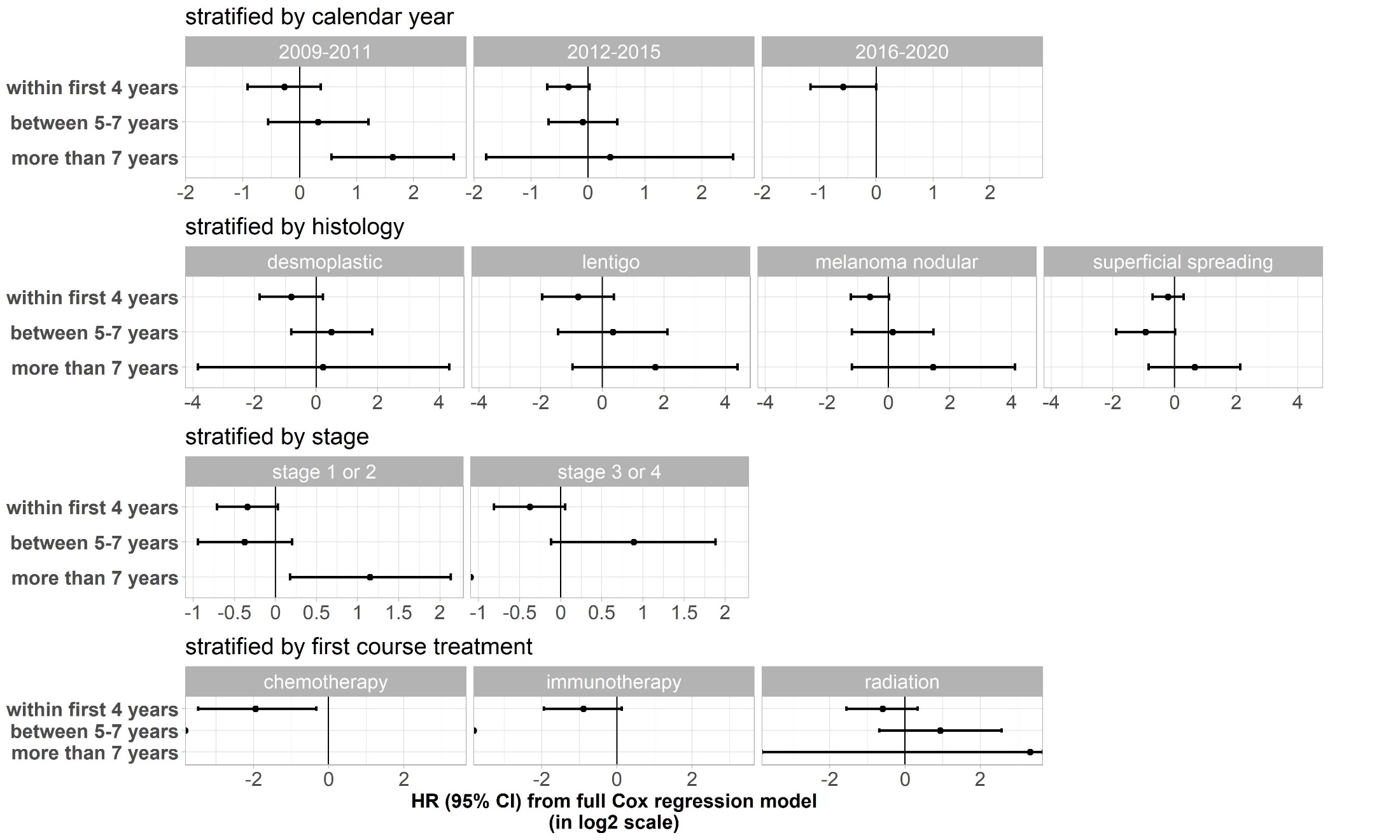

Supplement: Supplementary file 3 — Additional file 3. Association between history of keratinocyte carcinoma (KC) and survival following diagnosis of melanoma, stratified by patient characteristics.This figure depicts the association between history of KC and survival following melanoma among different patient groups as defined by calendar year of diagnosis, melanoma histology, stage of melanoma, and types of first course treatment. [file 12885_2023_11573_MOESM3_ESM.docx]
