# Supplementary material for: Melanoma and CLL co-occurrence and survival: role of KC history
Source: BMC Cancer. 2023 Nov 9;23:1084. doi: 10.1186/s12885-023-11573-z (PMC10636833; doi:10.1186/s12885-023-11573-z)
Supplement: Supplementary file 6 — Additional file 6. Association between history of keratinocyte carcinoma (KC) and survival following chronic lymphocytic leukemia (CLL) diagnosis among patients receiving specific chemotherapy/immunotherapy medications. This figure depicts the association between history of KC and survival following chronic lymphocytic leukemia among groups of patients as defined by the type of chemotherapy or the type of immunotherapy used as a part of first course treatment. [file 12885_2023_11573_MOESM6_ESM.docx]

**Additional file 6. Association between history of keratinocyte carcinoma (KC) and survival following chronic lymphocytic leukemia (CLL) diagnosis among patients receiving specific chemotherapy/immunotherapy medications.**

**
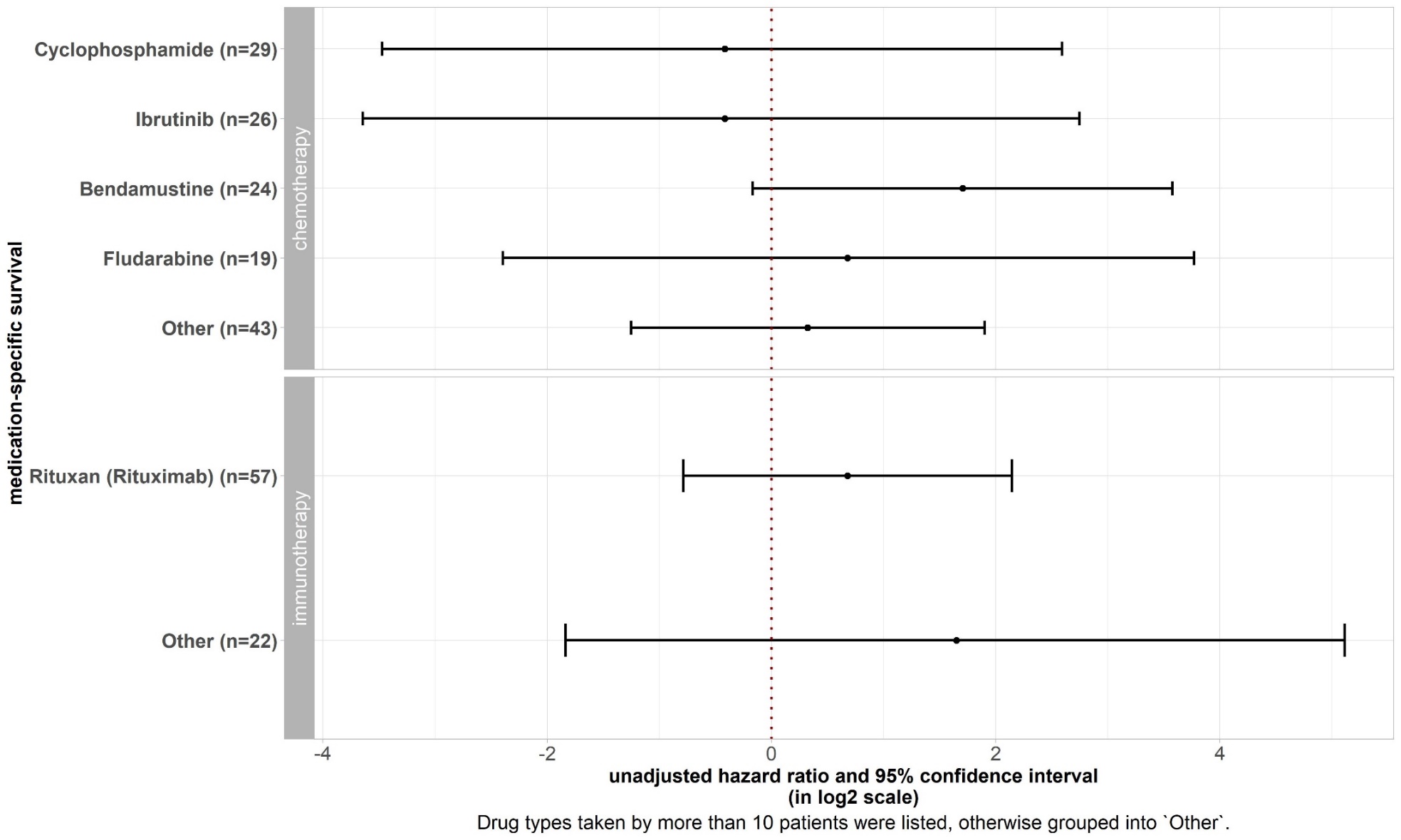
**
